# The role of the electrocardiographic phenotype in risk stratification for sudden cardiac death in childhood hypertrophic cardiomyopathy

**DOI:** 10.1093/eurjpc/zwab046

**Published:** 2021-03-27

**Authors:** Gabrielle Norrish, Cristian Topriceanu, Chen Qu, Ella Field, Helen Walsh, Lidia Ziółkowska, Iacopo Olivotto, Silvia Passantino, Silvia Favilli, Aris Anastasakis, Vasiliki Vlagkouli, Robert Weintraub, Ingrid King, Elena Biagini, Luca Ragni, Terrence Prendiville, Sophie Duignan, Karen McLeod, Maria Ilina, Adrian Fernández, Regina Bökenkamp, Anwar Baban, Fabrizio Drago, Peter Kubuš, Piers E F Daubeney, Sian Chivers, Georgia Sarquella-Brugada, Sergi Cesar, Chiara Marrone, Constancio Medrano, Reyes Alvarez Garcia-Roves, Orhan Uzun, Ferran Gran, Fernandez J Castro, Juan R Gimeno, Roberto Barriales-Villa, Fernando Rueda, Satish Adwani, Jonathan Searle, Tara Bharucha, Ana Siles, Ana Usano, Torsten B Rasmussen, Caroline B Jones, Toru Kubo, Jens Mogensen, Zdenka Reinhardt, Elena Cervi, Perry M Elliott, Rumana Z Omar, Juan P Kaski

**Affiliations:** 1 Centre for Inherited Cardiovascular Diseases, Great Ormond Street Hospital, Great Ormond Street, London WC1N 3JH, UK; 2 Institute of Cardiovascular Sciences, University College London, London, UK; 3 Department of Statistical Science, University College London, London, UK; 4 Department of Cardiology, The Children’s Memorial Health Institute, Warsaw, Poland; 5 Careggi University Hospital, Florence, Italy; 6 Cardiology Unit, A Meyer Pediatric Hospital, Florence, Italy; 7 Onassis Cardiac Surgery Center, Athens, Greece; 8 The Royal Children’s Hospital, Melbourne, Australia; 9 The Murdoch Children’s Research Institute; 10 University of Melbourne, Australia; 11 S. Orsola-Malpighi Hospital, Bologna, Italy; 12 Our Lady’s Children’s Hospital, Dublin, Ireland; 13 Royal Hospital for Children, Glasgow, UK; 14 Favaloro Foundation University Hospital, Buenos Aires, Argentina; 15 Leiden University Medical Center, Leiden, Netherlands; 16 Bambino Gesu Hospital, Rome, Italy; 17 University Hospital Motol, Prague, Czech Republic; 18 Royal Brompton and Harefield NHS Trust, London, UK; 19 Arrhythmia and Inherited Cardiac Diseases Unit, Hospital Sant Joan de Déu, University of Barcelona, Spain; 20 Medical Sciences Department, School of Medicine, University of Girona; 21 Papa Giovanni XXIII hospital, Bergamo, Italy; 22 Hospital General Universitario Gregorio Marañón, Madrid, Spain; 23 University Hospital of Wales, Cardiff, UK; 24 Val d’Hebron University Hospital, Barcelona, Spain; 25 University Hospital Virgen de la Arrixaca, Murcia, Spain; 26 Complexo Hospitalario Universitario A Coruña, CIBERCV, A Coruña, Spain; 27 John Radcliffe Hospital, Oxford, UK; 28 Southampton general Hospital, Southampton, UK; 29 Hospital Universitario Puerta de Hierro Majadahonda, CIBERCV, Madrid, Spain; 30 University Francisco de Vitoria, Pozuelo de Alarcon, Spain; 31 Department of cardiology, Aarhus University Hospital, Aarhus, Denmark; 32 Alder Hey Children’s hospital, Liverpool, UK; 33 Department of Cardiology and Geriatrics, Kochi Medical School, Kochi University, Japan; 34 Odense University Hospital, Odense, Denmark; 35 The Freeman Hospital, Newcastle, UK; 36 St Bartholomew’s Centre for Inherited Cardiovascular Diseases, St Bartholomew’s Hospital, West Smithfield, London, UK

**Keywords:** Children, Hypertrophic, Cardiomyopathy, Sudden death, Electrocardiogram

## Abstract

**Aims:**

The 12-lead electrocardiogram (ECG) is routinely performed in children with hypertrophic cardiomyopathy (HCM). An ECG risk score has been suggested as a useful tool for risk stratification, but this has not been independently validated. This aim of this study was to describe the ECG phenotype of childhood HCM in a large, international, multi-centre cohort and investigate its role in risk prediction for arrhythmic events.

**Methods and results:**

Data from 356 childhood HCM patients with a mean age of 10.1 years (±4.5) were collected from a retrospective, multi-centre international cohort. Three hundred and forty-seven (97.5%) patients had ECG abnormalities at baseline, most commonly repolarization abnormalities (*n* = 277, 77.8%); left ventricular hypertrophy (*n* = 240, 67.7%); abnormal QRS axis (*n* = 126, 35.4%); or QT prolongation (*n* = 131, 36.8%). Over a median follow-up of 3.9 years (interquartile range 2.0–7.7), 25 (7%) had an arrhythmic event, with an overall annual event rate of 1.38 (95% CI 0.93–2.04). No ECG variables were associated with 5-year arrhythmic event on univariable or multivariable analysis. The ECG risk score threshold of >5 had modest discriminatory ability [*C*-index 0.60 (95% CI 0.484–0.715)], with corresponding negative and positive predictive values of 96.7% and 6.7%

**Conclusion:**

In a large, international, multi-centre cohort of childhood HCM, ECG abnormalities were common and varied. No ECG characteristic, either in isolation or combined in the previously described ECG risk score, was associated with 5-year sudden cardiac death risk. This suggests that the role of baseline ECG phenotype in improving risk stratification in childhood HCM is limited.

## Introduction

The identification of individuals at increased risk of sudden cardiac death (SCD) is a cornerstone of clinical management in childhood hypertrophic cardiomyopathy (HCM). Paediatric-specific models, allowing clinicians to calculate individualized estimates of risk for the first time, have recently been developed, but their performance remains imperfect and additional predictors may be important for prognosis.^1^^,^^2^ The 12-lead electrocardiogram (ECG) is a routine, low cost clinical investigation in HCM that provides qualitative and quantitative information about the phenotype. The ECG phenotype of childhood HCM has not previously been systematically described, but abnormalities are seen in over 90% of adult patients.^3^^,^^4^ Studies in adults have reported conflicting findings about the association of individual ECG abnormalities [such as measures of left ventricular hypertrophy (LVH),^5^^,^^6^ abnormal repolarization pattern,^4^^,^^7^ or QT duration^8–10^] and SCD. However, to date, only a single group has investigated the role of ECG phenotype in risk stratification during childhood.^6^^,^^11^ An ECG risk score has been proposed to predict arrhythmic events in the HCM independently of traditional clinical risk factors, but this approach has not been independently validated in children.^11^^,^^12^ The aim of this study was to describe the ECG phenotype of childhood HCM in a large, international, multi-centre cohort and investigate its role in risk prediction for arrhythmic events.

## Methods

### Patient cohort

A multi-centre, retrospective cohort of patients aged 16 years or younger fulfilling diagnostic criteria for HCM with an available baseline resting 12-lead ECG were identified from the International Paediatric Hypertrophic Cardiomyopathy Consortium.^1^ Patients with HCM related to RASopathy, inborn errors of metabolism or neuromuscular disease, or a previous history of resuscitated cardiac arrest or sustained ventricular tachycardia were excluded. From the original consortium, an ECG was not available within 6 months of baseline evaluation for 437 patients and a further 92 patients were excluded due to poor ECG trace quality.

### Data collection

Anonymized, non-invasive clinical data were collected from baseline evaluation including: demographics, family history, symptoms (including heart failure symptoms defined as per New York Heart Association or Ross functional classification^13^), resting and ambulatory ECG, and two-dimensional, Doppler, and colour transthoracic echocardiography. Clinical risk factors for an arrhythmic event, as described in the HCM Risk-Kids model,^1^ were assessed at baseline, including: unexplained syncope; non-sustained ventricular tachycardia; body surface area-corrected maximal left ventricular wall thickness (MLVWT) *Z* score^14^; body surface area-corrected left atrial (LA) diameter *Z* score^15^; and maximal left ventricular outflow tract gradient (mmHg). Patients were routinely reviewed every 6–18 months at the discretion of the treating cardiologists. Data were collected independently at each collaborating centre.

### Electrocardiographic data features

Resting 12-lead ECGs from baseline evaluation were analysed using electronic callipers independently by four reviewers (G.N., E.F., C.T., or H.W.) unaware of the clinical details of the patients. Inter-observer reliability was quantified using the one-way intraclass coefficient and discrepancies were reviewed by a senior supervisor (J.P.K.). Age-specific normal values for ECG parameters were used.^16^ The following parameters were measured (average of 3 beats) from lead II, or V5 if quality of trace was poor: P wave amplitude (mV) and duration (ms), PR interval (ms), QRS axis, QRS duration (ms), QRS amplitude (mV), limb-lead QRS amplitude sum (mV), 12-lead amplitude-duration product^6^ (mV/s), QT interval (ms), corrected QT interval (ms) using Bazett’s formula,^17^ and Sokolow–Lyon score (SV1 or SV2 + RV5 or RV6 ≥35mm).^18^ The presence of the following parameters was described: left or right atrial enlargement, dominant S wave in V4, pathological Q waves, pathological T wave inversion (>1 mm beyond V1 aged ≥14 years, or beyond V3 aged <14 years), giant negative T waves (≥10 mm), giant positive T waves (≥10 mm), ST-segment depression (≥2 mm in any lead), ST-segment elevation (≥2 mm in leads V1–V3, or ≥1 mm in all other leads), left bundle branch block or right bundle branch block and three specific ECG patterns [‘pseudo-necrosis’, ‘pseudo-ST elevation myocardial infarction (pseudo-STEMI)’, and ‘low voltages’].^4^ Pseudo-STEMI was defined as the presence of Q waves ≥1/3 of the following R wave in depth and/or >0.04 s in duration in at least 2 contiguous leads except aVR and/or the lack of progressive R-wave voltage increase in the precordial leads.^4^ The ECG risk score, based on eight parameters (deviation in QRS axis, pathological T wave inversion in limb or precordial leads, ST-segment depression, dominant S wave in V4, limb-lead amplitude sum, 12-lead amplitude-duration product, and QTc), was calculated for all patients (Supplementary material online, *Table S1*).^6^

### Outcomes

The primary study end point was a composite outcome of major arrhythmic cardiac event (MACE), defined as SCD, resuscitated cardiac arrest, appropriate implantable cardioverter defibrillator (ICD) therapy for a ventricular tachyarrhythmia, or sustained ventricular tachycardia (VT) with haemodynamic compromise. The MACE end point was assessed at two time points: 5 years after baseline assessment and most recent evaluation prior to study end point (December 2017). Outcomes were determined by the treating cardiologist.

### Statistical analysis

MLVWT^14^ and LA diameter^15^ measurements are described in millimetres (mm) and as *z* scores relative to body surface area-corrected normal values. Continuous variables are described as mean (standard deviation) or median [interquartile range (IQR)], with two group comparisons made using Student’s *t*-test and Wilcoxon rank sum as appropriate. The distribution of categorical variables was compared using Chi-square test of Fisher’s exact test. Estimates of survival were obtained using the Kaplan–Meier product limit method.

Initially, univariable Cox regression models were used to screen a list of pre-specified variables (ECG and clinical) not included in the HCM Risk-Kids model or ECG risk score based on a significance level of 50% and complete case analysis. A multivariate model was then fitted with all the variables that had a *P*-value of <0.5 in the univariate screening together with all variables in the HCM Risk-Kids model and ECG risk score. The missing values for these variables were imputed using multiple imputation techniques based on chained equations.^19^ The imputation model included all explanatory variables, the Nelson Aalen estimator of the cumulative hazard function and the MACE outcome. A total of 40 imputed datasets were created. Penalized multivariable regression with least absolute shrinkage and selection operator was used to select the final variables for the multivariable model.

To assess the performance of the ECG risk score, a patient’s ECG risk score^6^ was calculated and a threshold score of >5 was used to define high risk, as described previously (Supplementary material online, *Table S1*). The discriminatory performance of using this threshold to identify patients at increased risk of MACE using an end point of 5-year follow-up and end of follow-up was determined using Harrell’s *C*-index. A value of 1 indicates perfect discrimination and a value of 0.5 indicates no discrimination.

The positive predictive value (PPV) of a threshold of >5 for experiencing an MACE at 5 years was calculated by dividing (sensitivity × prevalence) by [(sensitivity × prevalence) + (1 − specificity) × (1 − prevalence)] and expressed as a percentage. The negative predictive value (NPV) was calculated by dividing [specificity × (1 − prevalence)] by {(1 − sensitivity × prevalence) + [specificity (1 − prevalence)]} and expressed as a percentage.

Statistical analysis was performed in R (version 3.6.2).

### Ethics

Local ethical committee approval was obtained at each participating site. Informed consent was waived. The data underlying this article cannot be shared publicly as consent was not obtained for public dissemination of patient data.

## Results

### Baseline characteristics

A total of 356 patients with childhood HCM and available resting ECG data were identified from 28 centres from the HCM Risk-Kids cohort (*n* = 1029) (Supplementary material online, *Table S2*). Baseline demographic and clinical characteristics are described in *Table 1*. The ECG cohort had marginally less hypertrophy (mean MLVWT *Z* score 10.1 ± 6.5 vs. 11.1 ± 7.2; *P*-value 0.041) and were more likely to have a diagnosis made in the recent era [pre-2010 *n* = 125 (35.1%) vs. *n* = 578 (56.2%); *P*-value <0.001). Seventy-four patients (20.8%) had an ICD implanted during follow-up for primary (*n* = 70) or secondary (*n* = 4) prevention.

**Table 1:** Baseline demographic and clinical characteristics of Hypertrophic Cardiomyopathy Risk-Kids cohort and electrocardiogram cohort

Variable	HCM Risk-Kids (*n* = 1029)		ECG cohort (*n* = 356)		*P*-value
		Missing data, *n*		Missing data, *n*	
Male, *n* (%)	702 (68.2)	0	245 (68.9)	0	0.834
Pre-2000, *n* (%)	161 (15.6)	0	13 (3.7)	0	**<0.001**
2000–2010, *n* (%)	417 (40.5)		112 (31.4)		
2010 onwards, *n* (%)	451 (43.8)		190 (53.4)		
Age at baseline (years), mean ± SD	10.0 ± 4.5	0	10.1 ± 4.5	0	0.747
NYHA >1, *n* (%)	223 (22.1)	18	84 (23.6)		0.507
Family history of HCM, *n* (%)	534 (53.1)	23	196 (56.0)	6	0.378
Family history of SCD, *n* (%)	131 (12.8)	4	36 (10.1)	0	0.184
Unexplained syncope, *n* (%)	98 (9.6)	6	37 (10.4)	0	0.882
B-blocker therapy, *n* (%)	410 (40.2)	8	154 (43.3)	0	0.286
NSVT, *n* (%)	59 (6.9)	173	22 (7.1)	45	0.982
MWT (mm)	17.1 ± 7.4	86	16.4 ± 7	5	0.101
MWT *z* score, mean ± SD	11.1 ± 7.2	123	10.1 ± 6.5	12	**0.041**
LA diameter (mm), mean ± SD	32.7 ± 9.4	303	33.0 ± 8.8	65	0.726
LA diameter *z* score, mean ± SD	1.9 ± 2.3	354	1.82 ± 2.3	68	0.420
Maximal LVOT gradient (mmHg), median (IQR)	9 (6–22)	158	9.3 (6–20)	51	0.845

ECG, electrocardiography; HCM, hypertrophic cardiomyopathy; ICD, implantable cardiac defibrillator; IQR, interquartile range; LA, left atrium; LVOT, left ventricular outflow tract; MACE, major arrhythmic cardiac event; MWT, maximal wall thickness; NSVT, non-sustained ventricular tachycardia; NYHA, New York Heart Association; SCD: sudden cardiac death; SD, standard deviation. Bold Value Significance <0.05.

### Prevalence of electrocardiographic characteristics

**Figure 1:**
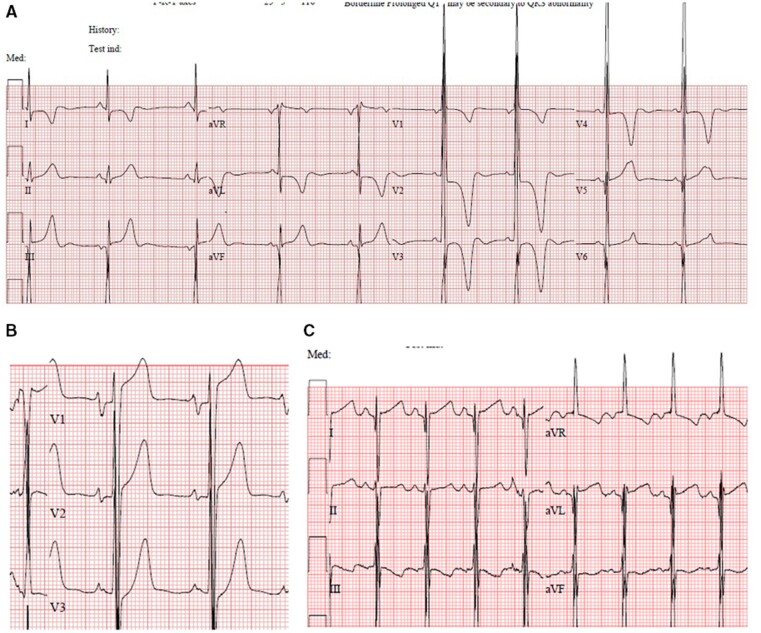
Examples of common electrocardiographic patterns. (*A*) Left ventricular hypertrophy, pseudo-necrosis, and T wave abnormalities. (*B*) Pseudo-ST elevation myocardial infarction pattern. (*C*) Right atrial enlargement, superior QRS axis, prolonged QT interval.

The prevalence of individual ECG abnormalities is described in *Table 2*. Nine patients (2.5%) had no ECG abnormalities at baseline. Two hundred and seventy-seven (77.8%) had one or more repolarization abnormalities. A pseudo-STEMI pattern was present in 93 patients (26.3%) [38 had ST elevation, 10 had giant positive T waves and 45 had both], a pseudo-necrosis pattern in 86 patients (24.4%) and a low QRS voltages pattern in 1 (0.3%). Examples of common ECG abnormalities are shown in *Figure 1*. One hundred and sixty-four patients (46.1%) had an ECG risk score of >5.

**Table 2:** Baseline electrocardiographic characteristics of the study population

ECG variable	Whole cohort (*n* = 356)	MACE (*n* = 25)	No MACE (*n* = 331)	*P*-value
QRS axis abnormal	126 (35.4%)	10 (40.0%)	116 (35.0%)	0.777
Left	87 (24.4%)			
Right	27 (7.6%)			
Extreme right/left	12 (3.4%)			
Pre-excitation	16 (4.5%)	1 (4.0%)	15 (4.5%)	>0.999
Left atrial enlargement (*n* = 354)	41 (11.6%)	2 (8.0%)	39 (11.8%)	0.416
Right atrial enlargement (*n* = 351)	70 (19.9%)	4 (16.0%)	66 (19.9%)	0.362
Pathological Q waves (*n* = 352)	167 (47.4%)	11 (44.0%)	156 (47.1%)	0.881
Inferior	84			
Lateral	14			
Inferolateral	66			
Anterior	3			
Giant inverted T waves (*n* = 352)	28 (8.0%)	4 (16.0%)	24 (7.3%)	0.132
Inferior	2			
Lateral	3			
Inferolateral	5			
Anterior	18			
Giant positive T waves (*n* = 353)	82 (23.2%)	9 (36.0%)	73 (22.1%)	0.186
Inferior	3			
Lateral	22			
Inferolateral	2			
Anterior	55			
Pathological T-wave inversion-any lead (*n* = 355)	196 (55.2%)	18 (72.0%)	178 (53.8%)	0.077
Limb leads (*n* = 355)	172 (48.5%)	17 (68.0%)	155 (46.8%)	0.069
Precordial leads (*n* = 353)	124 (35.1%)	14 (56.0%)	110 (33.2%)	**0.040**
ST-segment depression >2 mm	59 (16.6%)	6 (24.0%)	53 (16.0%)	0.398
Inferior	14			
Lateral	10			
Inferolateral	21			
Anterior	14			
ST elevation	122 (34.3%)	13 (52.0%)	109 (32.9%)	0.086
Inferior	25			
Lateral	17			
Inferolateral	16			
Anterior	64			
Dominant S wave in V4	152 (42.7%)	12 (48.0%)	140 (42.3%)	0.729
Sokolow–Lyon (mm) (*n* = 355)	46.4 (±29.7, range 6–42)	44.64 ± 20.20	46.58 ± 30.33	0.895
LVH (SLS ≥35 mm)	240 (67.7%)	17 (68.0%)	223 (67.4%)	0.965
Mean QRS duration (ms)	96.4 (±40.3)	100 ± 20	100 ± 40	0.321
QRS duration >120 ms	33 (9.3%)	3 (12%)	30 (9.1%)	0.625
QTc >440 ms	131 (36.8%)	13 (52.0%)	118 (35.6%)	0.102
Left bundle branch block	11 (3.1%)	1 (4.0%)	10 (3.0%)	>0.999
Right bundle branch block (*n* = 355)	9 (2.5%)	1 (4.0%)	8 (2.4%)	>0.999
ECG patterns				
Low QRS voltages (*n* = 355)	1 (0.3%)	0 (0.0%)	1 (0.3%)	>0.999
Pseudo-necrosis (*n* = 353)	86 (24.4%)	6 (24.0%)	80 (24.2%)	>0.999
Pseudo-STEMI (*n* = 353)	93 (26.3%)	11 (44.0%)	82 (24.8%)	0.065
Total ECG risk score	5.3 ± 3.4	6.6 ± 3.5	5.3 ± 3.4	0.057
Risk score >5	164 (46.1%)	16 (64.0%)	148 (44.7%)	0.062

ECG, electrocardiography; LVH, left ventricular hypertrophy; MACE, major arrhythmic cardiac event; STEMI, ST elevation myocardial infarction.

*N* = 356 unless otherwise indicated. Bold Value Significance <0.05.

### Arrhythmic events

Over a median follow-up of 3.9 years (IQR 2.0–7.7), 5 patients (1.4%) underwent cardiac transplantation and 14 (3.9%) died: SCD (*n* = 9, 2.5%), heart failure (*n* = 4, 1.1%), or thrombo-embolic event (*n* = 1, 0.3%). Overall annual mortality rate was 0.77 per 100 patient years (95% CI 0.46–1.30). Twenty-five patients had an MACE: appropriate ICD therapy *n* = 12 (3.4%), SCD *n* = 9 (2.5%), resuscitated cardiac arrest *n* = 3 (0.8%), and sustained VT with haemodynamic compromise *n* = 2 (0.6%). Overall annual MACE rate was 1.38 per 100 patient years (95% CI 0.93–2.04). ECG characteristics associated with MACE on univariable analysis are described in *Table 2*.

### Role of the electrocardiogram in predicting 5-year arrhythmic events

**Figure 2:**
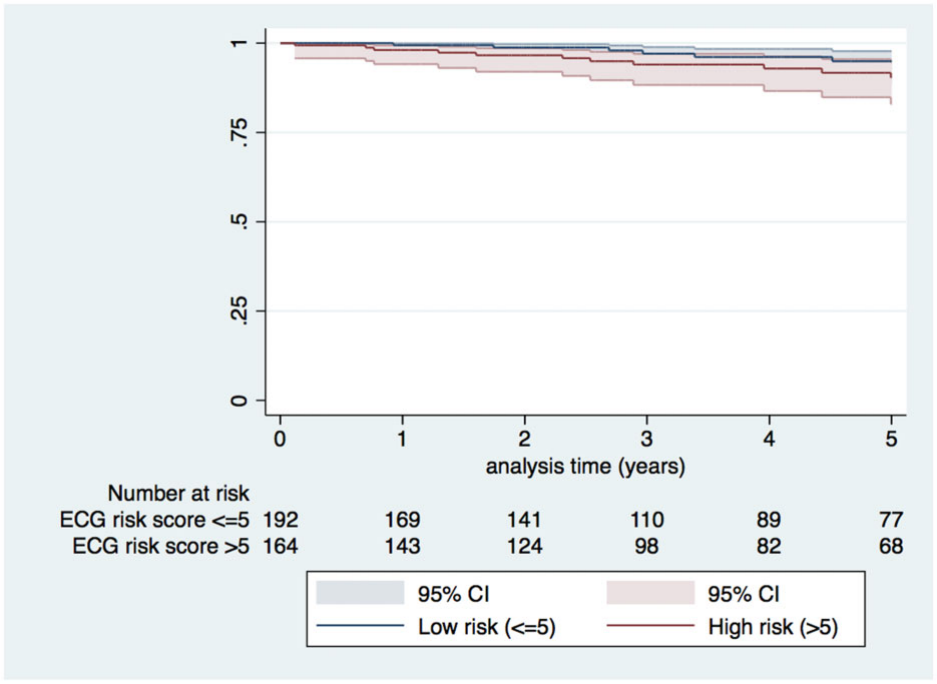
Kaplan–Meier survival curve showing event-free survival from major arrhythmic cardiac event by electrocardiography risk score.

In 17 patients, the MACE end point was reached within 5 years of follow-up. The clinical characteristics associated with 5-year MACE on Cox univariate regression analysis were measures of LVH [MLVWT HR 1.09 (95% CI 1.03–1.15, *P*-value 0.002), MLVWT Z score HR 1.07 (95% CI 1.01–1.13, *P*-value 0.002) and LA diameter HR 1.05 (95% CI 1.00–1.10, *P*-value 0.067)] (*Table 3*). No individual ECG variable, nor the ECG risk score, was associated with the end point (*Figure 2*). On multivariable analysis, only MLVWT, LVOT gradient and LA diameter were associated with MACE. MLVWT and LVOT gradient were selected in all imputed datasets by Lasso regression and LA diameter was selected in 60% of imputed datasets.

**Table 3:** Cox regression analysis for arrhythmic outcome within 5 years

Variable	Univariate Cox regression		Multivariate penalized regression
	HR (95% CI)	*P*-Value	Lasso estimates
Clinical risk factors			
Heart failure (NYHA >1)	0.62 (0.18–2.14)	0.446	
Family history of SCD	1.08 (0.25–4.73)	0.918	
Unexplained syncope	2.33 (0.76–7.14)	0.140	
NSVT	2.71 (0.77–9.53)	0.120	
MWT (mm)	1.09 (1.03–1.15)	**0.002**	1.063
MWT *z* score	1.07 (1.01–1.13)	**0.002**	
LA diameter (mm)	1.05 (1.00–1.10)	0.067	1.014
LA diameter *z* score	1.03 (0.84–1.26)	0.788	
Maximal LVOT gradient (mmHg)	1.00 (0.99–1.02)	0.519	1.001
ECG risk factors			
Pathological Q waves	0.65 (0.24–1.75)	0.394	
Giant inverted T waves	0.58 (0.77–4.39)	0.600	
Giant positive T waves	1.60 (0.59–4.33)	0.354	
Pathological T-wave inversion-any lead	1.85 (0.65–5.256)	0.247	
Pathological T-wave inversion limb leads	2.42 (0.85–6.88)	0.097	
Pathological T-wave inversion precordial leads	1.51 (0.58–3.90)	0.400	
ST-segment depression >2 mm	1.18 (0.67–2.07)	0.562	
ST elevation	2.06 (0.79–5.34)	0.138	
Dominant S wave in V4	1.46 (0.56–3.79)	0.436	
Limb-lead QRS sum (mV)	1.05 (0.97–1.14)	0.198	
Chest-lead QRS sum (mV)	1.00 (0.98–1.02)	0.950	
12-Lead QRS sum (mV)	1.00 (0.99–1.01)	0.849	
12-Lead product (mV)	1.03 (0.94–1.13)	0.576	
Sokolow–Lyon (mm)	1.00 (0.98–1.01)	0.939	
QTc >440 ms	1.51 (0.48–3.91)	0.400	
Left bundle branch block	1.73 (0.23–13.10)	0.595	
Right bundle branch block	1.96 (0.25–14.87)	0.515	
Low QRS voltages	0.00 (0, ∞)	0.998	
Pseudo-necrosis	0.89 (0.29–2.75)	0.846	
Pseudo-STEMI	2.37 (0.91–6.14)	0.076	
Total risk score	1.11 (0.97–1.28)	0.114	
Risk score >5	2.07 (0.77–5.60)	0.142	

ECG, electrocardiography; LA, left atrial; LVOT, left ventricular outflow tract; MWT, maximal wall thickness; NSVT, non-sustained ventricular tachycardia; NYHA, New York Heart Association; SCD, sudden cardiac death; STEMI, ST elevation myocardial infarction. Bold value Significance <0.05.

### Performance of electrocardiogram risk model in predicting 5-year arrhythmic event:

Of 164 patients with an ECG score >5, 153 (93.3%) did not have an MACE within 5 years and 147 (89.6%) did not have an event by the end of follow-up. Harrell’s *C*-index, which represents the probability of correctly distinguishing between high- and low-risk patients using an ECG risk score threshold of >5, was 0.60 (95% CI 0.484–0.715) at 5 years. The corresponding PPV and NPV were 6.7% (95% CI 4.7–9.4%) and 96.9% (95% CI 94.2–98.4%).

## Discussion

This study is the largest description of the ECG phenotype of childhood HCM published to date and shows a high prevalence of ECG abnormalities. One-third of patients did not meet ECG criteria for LVH, suggesting that it is not a sensitive measure of morphological LVH. No single ECG parameter was associated with risk. The ECG risk score had moderate discriminatory ability but with a low PPV. This suggests that the role of the baseline ECG phenotype in improving clinical risk stratification in childhood HCM is limited.

### Prevalence of electrocardiogram abnormalities in childhood hypertrophic cardiomyopathy

Less than 3% of the cohort had a normal resting 12-lead ECG, which is comparable to previous reports of 4–6% in adult patients.^3^^,^^4^ The most common ECG findings were repolarization abnormalities, LVH, QRS axis abnormalities and QT prolongation. Of note, one-third of patients did not meet ECG criteria for LVH, suggesting that this is not a sensitive measure of hypertrophy in childhood HCM as previously described in adult cohorts.^5^ One-third of patients had QT prolongation, which has previously been reported to be predictive of both all cause^10^^,^^20^ and arrhythmic mortality in adult HCM cohorts,^8^^,^^9^^,^^21^ but this does not appear to be the case in children. A small proportion of patients had ECG abnormalities typically associated with syndromic or metabolic disease (e.g. superior QRS axis and pre-excitation). As genotype information was not available, this raises the possibility of undiagnosed non-sarcomeric disease in these individuals.

Although the most common ECG abnormalities are similar to those reported in adult patients,^4^^,^^5^^,^^10^ age-specific differences were seen. Biagini *et al.*^4^ previously described three distinct ECG patterns in adult HCM patients: pseudo-necrosis, low QRS voltages, and pseudo-STEMI, which were more common in younger patients. In keeping with this, a higher proportion of our cohort had a pseudo-STEMI pattern (26% vs. 17%) whilst only one patient had low QRS voltages. The finding of low voltages in a small proportion (3%) of adult HCM patients did not appear to be related to end-stage disease and it is possible that differences in body habitus between paediatric and adult patients could explain the failure to observe this pattern during childhood. The proportion of patients with electrocardiographic evidence of LA enlargement was also lower than in adults (14% vs. 34%).^4^ The cardiac phenotype is recognized to evolve rapidly during childhood and early adulthood, and it is possible that these differences reflect age-related progression of both the cardiac and electrocardiographic phenotype. Further studies correlating the evolving ECG and cardiac phenotype in childhood HCM are required.

Finally, ECG abnormalities have been reported to be present in a proportion of genotype-positive phenotype-negative patients prior to the development of LVH^22^^,^^23^ and ECG screening has been proposed to screen for cardiac disease in young athletes.^24^ It is beyond the scope of this study to assess the yield of ECG screening for childhood HCM, but future studies designed to investigate this would be helpful.

### The association of electrocardiogram abnormalities with arrhythmic events

Children are known to have a higher overall risk of arrhythmic events compared to adult patients (1.1 vs. 0.8 per 100 patient years),^1^^,^^25–27^ despite a lower proportion of traditional clinical risk factors, suggesting that additional risk factors may be important in childhood HCM. Previous studies, all but one of which have included mainly adult patients, have reported conflicting findings regarding the association of individual ECG abnormalities and arrhythmic events.^4–6^^,^^9^^,^^10^ The only previous paediatric study reported a significant association between an arrhythmic event and individual ECG abnormalities [such as LVH (sum R/S waves, Sokolow–Lyon index and QRS amplitude-duration product) and ST-segment depression].^11^^,^^12^ In contrast, in this study, although a higher proportion of patients with T wave inversion experienced an MACE, no individual ECG abnormalities at baseline were statistically associated with 5-year risk of an arrhythmic event on time-dependent univariable or multivariable analysis. As previous studies have shown that ECG patterns may correlate only weakly with a patient’s clinical phenotype,^5^ it is perhaps not surprising that ECG variables on their own have a limited ability to predict risk. On multi-variable analysis, only clinical risk factors previously recognized to be associated with SCD (e.g. measures of LVH, LA dilatation and left ventricular outflow tract obstruction), and included in current risk stratification guidelines, were associated with the arrhythmic end point. The results suggest that individual ECG parameters may not improve current risk prediction models.

### The performance of the electrocardiogram risk score

The ECG risk score reported by Ostman-Smith *et al.* was initially developed in a small cohort of adult patients and has been reported to be a strong predictor of SCD in children with HCM,^6^^,^^12^ with a score of >5 having a PPV and NPV of 45% and 99% for an arrhythmic event, respectively.^12^ The present study represents the first external validation of the ECG risk score in childhood and shows it to have a modest ability to discriminate between high- and low-risk patients over 5 years follow-up. This performance is comparable to the current paediatric ESC^28^/AHA guidelines,^29^ but lower than the recently published paediatric-specific risk models,^1^^,^^2^ and supports the hypothesis that, although individual ECG parameters may not be predictive of events, a composite score could be useful. The high NPV of the ECG risk score means that patients with a low score could indeed be reassured. However, the low PPV (<10%) suggests that, if used on its own to guide ICD implantation, the majority of patients classified as high risk would not experience an appropriate therapy but would be exposed to the risk of long-term device-related complications.^30^ Indeed, over two-fifth of our cohort had an ECG score of >5 and would have been defined as high risk leading to ICD implantation. Importantly, the baseline ECG score was not associated with arrhythmic events when traditional clinical risk factors were accounted for suggesting that its role in improving risk stratification may be limited.

The poorer performance of the ECG risk score in this cohort may in part be explained by the timing of ECG recordings or length of follow-up. Although the ECG risk score was developed using ECGs from baseline assessment, in the previous paediatric validation study, the last available ECG before an event or end of follow-up was used meaning the calculated ECG risk score was temporally related to the event.^12^ This likely reflects an evolving cardiac phenotype during childhood and future studies exploring the use of serial clinical investigations for predicting risk should include ECG parameters. The outcome of 5-year MACE was chosen to assess the ability of the ECG risk score to predict events that could be treated by ICD implantation during childhood as the reported longevity of an ICD device is between 5 and 7 years.^31^ Nonetheless, the performance of the model did not improve when the whole follow-up was considered (Supplementary material online, *results*).

### Limitations

HCM is a rare disease in childhood and this study is limited by small numbers of patients and events, despite being recruited from a large international consortium of expert centres (*n* = 39). Due to its retrospective design, only one-third of the HCM risk SCD cohort had a high quality 12-lead ECG from within 6 months of baseline assessment and this differed by era. Fading of ECG traces over time is well-recognized and until recently ECGs were not routinely digitally stored posing a challenge for retrospective studies. A larger number of historic patients (pre-2010) did not meet inclusion criteria but the mean length of follow-up in the ECG or whole cohort did not differ. The reason for diagnosis for the included patients is not known, but they had a marginally lower mean MLVWT, which could be explained by a higher proportion of screening patients included in more recent eras. The cohort did not otherwise differ in terms of baseline clinical characteristics, including family history of HCM, and incidence of arrhythmic events to other large population studies. This suggests that the results of this study are representative and applicable to a wider childhood HCM population. Nonetheless, the small number of events may have limited our ability to detect statistically significant differences. This cohort of patients did not include those presenting in infancy or with syndromic disease who are known to have a worse prognosis and may differ in ECG phenotype. Future studies describing and investigating the role of the 12-lead ECG in these patient groups are needed.

## Conclusions

In a large, international, multi-centre cohort of childhood HCM, ECG abnormalities were common and varied, occurring in over 95% of patients. Despite a high prevalence of abnormalities, no individual ECG findings were associated with an arrhythmic event. The ECG risk score had a modest ability to discriminate between high- and low-risk patients but with a low PPV. This suggests that the role of baseline ECG phenotype in improving risk stratification in childhood HCM is limited.

## Supplementary material

Supplementary material is available at *European Heart Journal – Cardiovascular Pharmacotherapy* online.

## Supplementary Material

zwab046_Supplementary_Information

## Notes

Centre for Inherited Cardiovascular Diseases, Great Ormond Street Hospital; Arrhythmia and Inherited Cardiac Diseases Unit, Hospital Sant Joan de Déu, University of Barcelona; Hospital Universitario Puerta de Hierro Majadahonda, CIBERCV; and St Bartholomew’s Centre for Inherited Cardiovascular Diseases, St Bartholomew’s Hospital are the members of ERN GUARD-HEART (European Reference Network for Rare and Complex Diseases of the Heart; http://guardheart.ern-net.eu).
