# Supplementary material for: The role of the electrocardiographic phenotype in risk stratification for sudden cardiac death in childhood hypertrophic cardiomyopathy
Source: Eur J Prev Cardiol. 2021 Mar 27;29(4):645–53. doi: 10.1093/eurjpc/zwab046 (PMC8967480; doi:10.1093/eurjpc/zwab046)
Supplement: zwab046_Supplementary_Information [file zwab046_supplementary_information.docx]

**Supplementary table 1: ECG risk score**

| **ECG parameter** | | **Number of points** |
| --- | --- | --- |
| Any deviation in QRS axis | | 1 |
| Pathological T-wave inversion limb leads | | 1 |
| Pathological T-wave inversion precordial leads* | | 2 |
| ST-segment depression ≥2mm | | 2 |
| Dominant S wave in V4 | | 2 |
| Limb-lead QTS-amplitude sum (mV) | ≥7.7 | 1 |
|  | ≥10.0 | 2 |
|  | ≥12.0 | 3 |
| 12-lead amplitude-duration product (mV/sec) | ≥2.2 | 1 |
|  | ≥2.5 | 2 |
|  | ≥3.0 | 3 |
| QTc | | 1 |
|  | | **Maximum score = 14** |

*The two points for precordial T-wave inversion does not get added on top of the

1 point for limb-lead T-wave inversion, thus total score available for T-wave

abnormalities is 2 points.

Adapted from Ostman-Smith et al^6^

Supplementary table 2: Number of patients in ECG cohort per centre

|  | **Centre** | **Number of patients enrolled** | **% of cohort** |
| --- | --- | --- | --- |
| **1** | Great Ormond Street Hospital, London, UK | 89 | 25.0 |
| **2** | Children’s Memorial Health Institute, Warsaw, Poland | 72 | 20.2% |
| 3 | Our Lady’s Children’s Hospital, Dublin, Ireland | 28 | 7.9% |
| **4** | Hospital Sant Joan de Deu, Barcelona, Spain | 28 | 2.8% |
| **5** | Careggi University Hopsital, Florence, Italy | 24 | 6.7% |
| **6** | S. Orsola-Malpighi Hospital, Bologna, Italy | 19 | 5.3% |
| **7** | The Royal Children’s Hospital, Melbourne, Australia | 14 | 3.9% |
| **8** | Royal Brompton and Harefield NHS Trust, London, UK | 12 | 3.4% |
| 9 | Onassis Cardiac Surgery Centre, Athens, Greece | 11 | 3.1% |
| **10** | Favaloro Foundation University Hospital, Buenos Aires, Argentina | 10 | 2.8% |
| **11** | University Hospital Motol, Prague, Czech Republic | 10 | 2.8% |
| **12** | Bambino Gesu Hospital, Rome, Italy | 6 | 1.7% |
| **13** | Val d’Hebron University Hospital, Barcelona, Spain | 6 | 1.7% |
| **14** | Complexo Hospitalario Universitario A Coruña, Spain | 6 | 1.7% |
| **15** | Hospital Universitario Puerta de Hierro Majadahonda Madrid, Spain | 5 | 1.4% |
| **16** | University Hospital of Wales, Cardiff, UK | 4 | 1.1% |
| **17** | Southampton General Hospital, Southampton, UK | 4 | 1.1% |
| **18** | Aarhus University Hospital, Aarhus, Denmark | 3 | 0.8% |
| **19** | Hospital General Universitario Gregorio Maranon, Madrid, Spain | 3 | 0.8% |
| **20** | Leiden University Medical Center, Leiden, Netherlands | 3 | 0.8% |
| **21** | Alder Hey Children’s hospital, Liverpool, UK | 3 | 0.8% |
| **22** | Kochi Medical School Hospital, Kochi University, Japan | 3 | 0.8% |
| **23** | Odense University Hospital, Odense, Denmark | 3 | 0.8% |
| **24** | Freeman Hospital, Newcastle, UK | 2 | 0.6% |
| **25** | Royal Hospital for Children, Glasgow, UK | 2 | 0.6 |
| 26 | University Hospital Virgen de la Arrixaca, Murcia, Spain | 2 | 0.6% |
| **27** | Papa Giovanni XXIII hospital, Bergamo, Italy | 1 | 0.3% |
| **28** | John Radcliffe Hospital, Oxford, UK | 1 | 0.3% |

Centres in the IPHCM consortium who did not contribute ECG traces for this study include;

Monaldi Hospital, Naples, Italy; Leeds General Infirmary, Leeds, UK; Bristol Royal hospital for Children, Bristol, UK; Niguarda Hospital, Milan, Italy; University Hospital La Paz, Madrid, Spain; Glenfield Hospital, Leicester, UK; University Hospitals Parma, Italy; Ghent University Hospital, Belgium; Mater Dei Hospital, Malta; Evelina Children’s Hospital, London, UK; Birmingham Children’s Hospital, Birmingham, UK

Supplementary results

*Performance of ECG risk model in predicting arrhythmic events occurring during follow up:*

Of 164 patients with an ECG score >5; 148 (90.2%) did not have a MACE event by the end of follow up. Harrell’s C-index, which represents the probability of correctly distinguishing between high and low risk patients using an ECG risk score threshold of >5 was 0.610 (95% confidence interval 0.484-0.722). The corresponding positive and negative predictive values were 9.8% (95% CI 7.3-13.0%) and 95.3% (95% CI 92.3-97.2%).
